# Vitamin C variation in citrus in response to genotypes, storage temperatures, and storage times: A systematic review and meta-analysis

**DOI:** 10.1016/j.heliyon.2024.e29125

**Published:** 2024-04-10

**Authors:** Rahmat Budiarto, Syariful Mubarok, Mohammad Miftakhus Sholikin, Dwi Novanda Sari, Ana Khalisha, Stefina Liana Sari, Bayu Pradana Nur Rahmat, Tri Ujilestari, Danung Nur Adli

**Affiliations:** a Department of Agronomy, Faculty of Agriculture, Universitas Padjadjaran, Sumedang 45363, Indonesia; b Research Center for Animal Husbandry, Research Organization of Agriculture and Food, National Research and Innovation Agency (BRIN), Bogor 16915, Indonesia; c Study Program of Agrotechnopreneur, Faculty of Agriculture, Universitas Padjadjaran, Sumedang 45363, Indonesia; d Graduate School of Life and Environmental Science, University of Tsukuba, Tsukuba, Japan; e Research Center for Food Technology and Processing, National Research and Innovation Agency (BRIN), Gunungkidul 55861, Indonesia; f Faculty of Animal Science, Universitas Brawijaya, Malang, 65145, Indonesia; g Meta-Analysis in Plant Science (MAPS) Research Group, Bandung 40621, Indonesia; h Animal Feed and Nutrition Modelling Research Group, IPB University, Cibinong 16911, Indonesia; i Center for Tropical Animal Studies (CENTRAS), The Institute of Research and Community Empowerment of IPB (LPPM IPB), Bogor 16680, Indonesia

**Keywords:** Citrus fruit, Meta-analysis, Storage time, Storage temperature, Vitamin C

## Abstract

Numerous published studies have highlighted discrepancies in the duration and storage temperature used for preserving vitamin C content on various citrus genotypes worldwide. The present study aimed to analyze the variation in vitamin C content as influenced by citrus genotype, duration, and storage temperature using meta-analysis approaches. Data searching, selection, and tabulation resulted in a comprehensive database constructed from 1412 data points gathered from 54 individual studies, following PRISMA-P guidelines. The vitamin C content varied widely, ranging from 0 to 76.2 mg/100 mL in whole data of citrus fruit. Meta-analysis findings revealed that the duration of storage significantly impacted the vitamin C content in citrus fruits. Specifically, for grapefruit, mandarin, and orange, the length of storage significantly influenced their vitamin C levels (*P* < 0.01), with a consistent decrease observed over time. The correlation coefficients (R^2^) were 0.63 for grapefruit, 0.9 for mandarin, and 0.69 for orange. In contrast, no significant difference was found in terms of vitamin C levels between hybrid and lime citrus concerning the impact of storage time. However, other results indicated a significant influence of storage temperature on the variation in vitamin C levels for both citrus and hybrid varieties (*P* < 0.001). Depending on the genotype, tangerine had significantly lower vitamin C content compared to other varieties, at 16.9 mg/100 mL, with vitamin C ranging from 13.2 to 20.9 mg/100 mL (*P* < 0.001). The highest vitamin C content was found in lemon and hybrid varieties, around 65.5 (range 59.3–76.2) and 48.3 (range 29.6–75.5), respectively, all in mg/100 mL (*P* < 0.001). Furthermore, there was a tendency for decreasing vitamin C concentration due to temperature (*P* = 0.078), while citrus variety experienced a decrease, although not significant. The ideal temperature (15 °C) and duration of storage (56 days) to minimize vitamin C loss in citrus fruits are at their optimum point. In conclusion, the deterioration of vitamin C in citrus fruits is influenced by both temperature and storage duration, and its content is also impacted by the variety of citrus.

## Highlights

• A meta-Analysis come from 55 studies and 1402 points, this study explores vitamin C variation in citrus.
• Significant impacts on vitamin C levels, particularly for grapefruit, mandarin, and orange varieties.
• Suggesting an ideal storage of 15 °C for 56 days to maintain stable vitamin C at 60.2 mg/100 mL.

## Graphical abstract

Summary of a meta-analysis on variations in vitamin C concentration caused by storage duration and temperature conditions.

**Figure:**
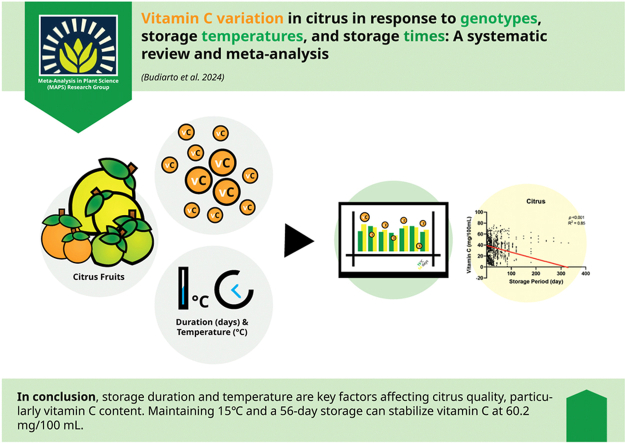

## 1 Introduction

Citrus stands as a lucrative, widely recognized, and significant horticultural crop globally [1]. Recent statistics indicate a cultivation area of approximately 10.1 million hectares and an annual production of 159 million tons [2]. Before becoming a worldwide popular commodity, citrus is believed to have originated from certain places in Asia [[3], [4], [5]]. The main reason behind citrus popularity is its delicious and nutritious fruit [6,7] containing various beneficial phytochemicals [8,9]. One of the noticeable bioactivities of citrus is its role as an antioxidant [10,11], so consuming citrus can be associated with maintaining the immune system and body health [12,13]. Immunity and healthy lifestyles have been extensively discussed recently due to the COVID-19 outbreak [14,15], and thus, consuming nutritious fruit such as citrus become very important.

The most popular nutrient within citrus fruits is vitamin C [16,17] thus, citrus consumption is believed to be an effective strategy to meet the daily need for vitamin C [18]. Numerous studies report the role of vitamin C as the major antioxidant in citrus fruits [[19], [20], [21], [22], [23]]. Vitamin C in citrus is higher than in other fruits, such as banana, grape, rose apple, and soursop [24], except guava, with a range of 140–146 mg per 100 g [25]. In addition, assessing the nutritional quality of citrus can also refer to its vitamin C content [[26], [27], [28], [29], [30], [31]]. If vitamin C is well preserved, then most of the other nutrients are well preserved [32]. In contrast, the decomposition of vitamin C relates to the reduction of beneficial phytochemical content, such as hesperidin [33].

Vitamin C is one of several vitamins contained in citrus and its derivative products [34]. This vitamin acts as a strong antioxidant [20,35], to overcome problems related to oxidative stress [36], mainly detoxifying free radicals and reactive oxygen species [37]. In addition, this vitamin also accelerates body recovery, against viral infections, such in cases of malaria [38], influenza [39], and COVID-19 [40]. A weakened immune condition, leading to severe illness occurrence, is believed to be associated with vitamin C deficiency [41]. Therefore, vitamin C-rich citrus consumption is required and should be formulated.

The formulation of vitamin C-rich citrus is feasible to carry out by employing certain modifications in both pre- and post-harvest periods. During pre-harvest period, the selection of both suitable growing locations and demandable citrus genotypes with high vitamin C content are urgently needed. The content of vitamin C is highly dependent on both genetics [31,42,43], and environment [31,44]. Concerning genetics, different genotypes may show different content of vitamin C, as previously reported by numerous researchers, both at inter- and intra-species levels [[45], [46], [47], [48]]. Vitamin C content is likely multigenic inheritance [45], thus multiple genes are involved in vitamin C biosynthesis [49]. Concerning environmental factor, different plant growing locations, implying the variation of climatic, edaphic, and applied culture practice, may result in different vitamin C content. In addition to pre-harvest, the post-harvest modification is also important aspect to study for maintaining the vitamin C content until it reaches consumer. Storage became one of several important treatments frequently applied in post-harvest period. The longer storage time, the higher vitamin C loss [50,51]. In addition, temperature of storage is also urgently studied due to its major effect on vitamin C content [18,52]. Higher storage temperature results in higher vitamin C loss and a shorter shelf-life period [53,54]. Thus, determination of the optimal range of storage temperature and storage time is required to obtain better vitamin C retention in the citrus product [21].

Numerous published studies show the discrepancy in storage time and temperature used for the preservation of various citrus genotypes worldwide. However, there are still limited studies that can summarize all of this quantitatively, to gain a greater understanding of the magnitude of genotype, origin, and storage effect on citrus vitamin C. For instance, as noted in previous study [55], researchers reported the vitamin C content in fruits and vegetables, yet they did not specify details such as specific fruits like citrus and their various strains. Conversely, another study reported a limited efficacy of vitamin C as an intervention for human blood pressure [56]. Meta-analysis is currently garnering widespread attention due to its feasibility for extracting and interpreting results from big data more efficient and evidence-based [57,58]. Therefore, the present study aims to analyze the variation of vitamin C as influenced by citrus genotype and storage conditions (i.e., duration and temperature), using meta-analysis approaches.

## 2 Materials and methods

### 2.1 Article search

Initial step to construct a database was conducted from 1948 to present. A database was composed of some published scientific reports in the form of journal article, excluding review paper. Those articles were collected from including Web of Science, Science Direct, Scopus, and Google Scholar. Several specific keywords used were as follows: “citrus” [MeSH Terms] OR “citrus fruits” [All fields] OR “citrus fruits” [All Fields]) AND (“vitamin C” [MeSH Terms] OR “ascorbic acid” [All Fields]) AND (“storage” [MeSH Terms] OR “storage duration” OR “storage temperature” [All Fields]) resulting 525, 247, 111, and 260 published studies, respectively. In the reference manager, the selection process was carried out by considering the relevance of studies to the predetermined research topics, i.e., citrus vitamin C in response to genotypes and storage treatment.

### 2.2 Eligibility criteria, selection process, and data extracted

The selection process aimed to ensure the inclusion of only pertinent and indexed papers in the database. Articles were chosen based on specific criteria: (1) they had to be from single studies indexed by platforms such as Google Scholar, with a digital object identifier (DOI) or a globally accessible uniform resource locator (URL); (2) they had to report information on vitamin C variation in citrus and its correlation with storage treatment; and (3) the selected journals needed to present quantitative data on vitamin C across various temperatures and storage times, both in tabular and graphical formats.

The strength of the present meta-analysis lay in providing clearer patterns of mediators (genotype, storage time, and storage temperature) effect on vitamin C, leading to the generalizability of results and allowing more accurate and higher statistical power estimation of vitamin C. This was particularly crucial given conflicting results from individual studies, such as the ideal temperature at 15 °C and the duration of storage at 56 days. Meanwhile, a limitation of the present meta-analysis was the absence of relative humidity (RH) data. Temperature control alone proved insufficient; therefore, humidity regulation becomes a crucial compliment factor. A lower RH levels could lead to increased transpiration rates and a faster deterioration in external fruit quality.

Article evaluation was made simpler by utilizing the PICO (population, intervention, comparison, and outcomes) framework, along with the study selection criteria detailed. In this framework, the population was vitamin C, the intervention was citrus, the comparison was storage, and the main outcomes included storage time (day), storage temperature (°C), and vitamin C content (mg/100 mL). Meanwhile, the general exclusion criteria from the population were fruits that didn't report the vitamin C. Then, the intervention exclusion criteria involved changes in conditions that might have caused fluctuations, necessitating additional treatments like chemical, biological, or physical methods, which could directly affect vitamin C levels. As for outcome exclusion criteria, variations in citrus fruit vitamin C concentration due to factors unrelated to storage temperature and duration were excluded, along with qualitative value representations. Additionally, review articles, theses, book chapters, and non-English *in vitro* studies were excluded based on study design consistency.

The initial assessment included 427 peer-reviewed publications that discussed variations in vitamin C levels in citrus. Of these, 138 publications were deemed ineligible and did not meet the criteria. Additionally, 116 publications were excluded due to unavailability of records. Another 34 articles were excluded as they were not statistical and were solely reviews. Five articles lacked a digital object identifier (DOI), and 11 articles were excluded because they were not consistently in English, with four using the same data. Ultimately, 65 peer-reviewed data were excluded as they reported non-experimental studies (see Table 1).

**Fig. 1:**
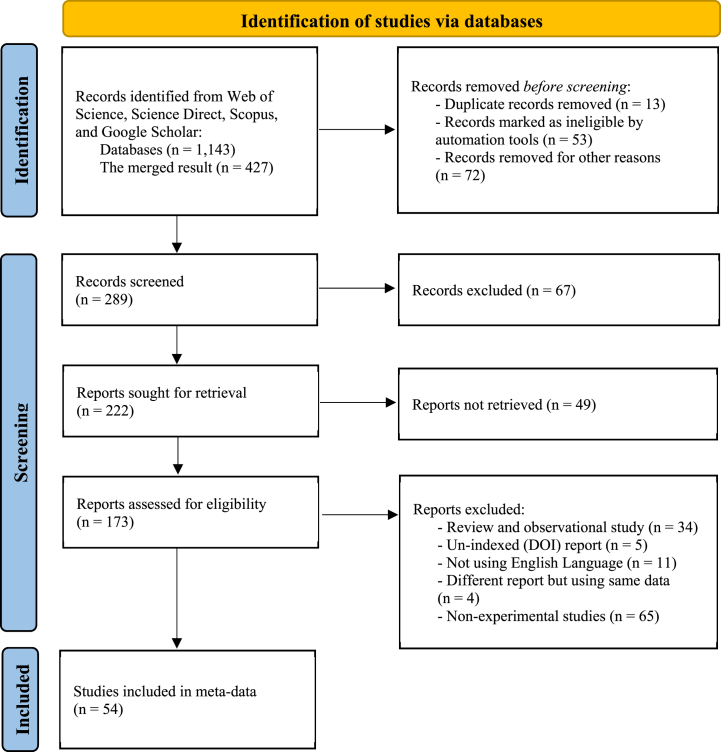
Illustrates the PRISMA-P Diagram outlining the manuscript selection process for conducting a meta-analysis on the vitamin C content in citrus fruits, considering different storage durations and temperature conditions.

From the post-selected 54 single studies, a database was constructed, as outlined in Table 2. Each study contributed variables such as citrus origin, citrus species, citrus storage time, citrus storage temperature, and publication year. A total of 1412 data points on vitamin C were extracted for further statistical meta-analysis. While most cases reported vitamin C units as mg/100 mL, unit conversion was carried out in certain studies to adhere to international standards. The present meta-analysis followed the preferred reporting items for systematic review and meta-analysis protocols (PRISMA-P), depicted in Fig. 1 [59,60], similar to a prior study on kaffir lime meta-analysis [61].

**Table 1:** PICO and study design criteria inclusion and exclusion of studies.

Parameters	Inclusion criteria	Exclusion criteria
Population	Citrus fruits that contain vitamin C (subject)	Fruits other than citrus that provide vitamin C
Intervention	The presence of vitamin C in citrus fruits is affected by factors such as storage duration and temperature	Changes in these conditions can lead to variations, prompting the need for additional treatments such as chemical, biological, or physical methods, which can directly impact the vitamin C content
Comparison	Temperature conditions and storage duration	No temperatures or durations reported
Outcomes	The fluctuation of vitamin C levels due to temperature and duration of storage in citrus fruits	The variation in vitamin C concentration in citrus fruits due to factors other than temperature and duration of storage, as well as the representation of qualitative values
Study design	Recorded were experimental-random studies on the temperature and duration of storage of citrus fruits	Review articles, theses, book chapters, and *in vitro* studies that are not consistently written in the English language

**Table 2:** Database for meta-analysis of vitamin C content in citrus. It includes information on citrus origin, citrus species, storage time, and storage temperature, referencing the relevant sources for each entry.

Citrus origin	Citrus species	Citrus fruit storage		Ref.	Study
		Period	Temperature		
Amambai, Brazil	*Champagne orange*	0–16 days	5 °C	[64]	S1
Antalya, Turkey	*Clementines mandarins*	20, 40, & 60 days	1 °C	[65]	S2
Antalya, Turkey	*Valencia oranges*	60, 120, & 180 days	4 °C	[66]	S3
Araraquara, Brazil	*Valencia oranges*	6 days	24 °C	[67]	S4
California, US	*Navel oranges*	0, 15, 30, 37, & 45 days	3 °C	[68]	S5
Corrientes, Argentina	*Duncan grapefruit*	0, 5, 10, 15, 20, 25, & 30 days	4 °C	[69]	S6
Daegu, South Korea	*Daegu mandarin*	0, 5, 10, & 15 days	4 °C	[70]	S7
Darab, Iran	*Key acid lime*	35 days	10 & 20 °C	[71]	S8
Ganzhou, China	*Navel oranges*	0, 15, 30, 45, 60, 75, 90, 105, & 120 days	–	[72]	S9
Guangdong, China	*Mandarin*	28 & 56 days	6 & 25 °C	[73]	S10
Hachula valley, Israel	*Star ruby grapefruit*	0–112 days	2 & 11 °C	[74]	S11
Hangzhou, China	*Satsuma mandarin*	0, 3, 6, 9, & 12 days	4, 10, & 20 °C	[54]	S12
Hatay, Turkey	*Nova mandarin*	0, 15, 30, 45, 60, 75, 90, 105, & 120 days	4 & 6 °C	[75]	S13
Ismailia, Egypt	*Valencia orange &* *Navel orange*	20, 40, & 60 days	1, 10, & 20 °C	[76]	S14
Jabalpur, India	*Kagzi lime*	6–18 days	25–30 °C	[77]	S15
Jabalpur, India	*Kagzi lime*	6–24 days	25–30 °C	[78]	S16
Jaguaribe, Brazil	*Delta valencia orange*	0, 4, 8, 12, 16, 20, 24, & 28 days	7 °C	[79]	S17
Khartoum, Sudan	*Sinnari green oranges*	0–15 days	25–30 °C	[80]	S18
Khuzestan, Iran	*Valencia orange &* *Siavarz orange*	30–90 days	6 °C	[81]	S19
Lake Placid, US	*Valencia orange*	2 & 32 days	16 °C	[82]	S20
Mersin, Turkey	*Turkish mandarin*	0–27 days	25 °C	[83]	S21
Nagpur, India	*Nagpur mandarin,* *Mosambi orange,* *Kagzi acid lime*	0, 15, 30, 45, & 75 days	6–7 °C (mandarin & oranges) & 8 °C (lime)	[84]	S22
Neergabby, Australia	*Eureka lemon*	30, 60, & 90 days	10 °C	[85]	S23
Oristano, Italy	*Malvasio mandarin*	At harvest; 42 & 84 days	4 °C	[86]	S24
Peshawar, Pakistan	*Oranges*	0, 15, 30, & 45 days	25–30 °C	[87]	S25
Porto Alegre, Brazil	*Montenegrina &* *Rainha tangerines*	7 days	20 °C	[88]	S26
Poznan, Poland	*Oranges*	0, 60, 120, & 180 days	18, 28, & 38 °C	[89]	S27
Punjab, India	*Kinnow mandarin*	30–65 days & 5–15 days	5–19 °C	[90]	S28
Punjab, India	*Kinnow mandarin*	5, 10, 15, 20, & 25 days	18–20 °C	[91]	S29
Punjab, India	*Kinnow mandarin*	5, 10, 15, 20, & 25 days	18–20 °C	[92]	S30
Punjab, Pakistan	*Local oranges*	0, 10, 20, 30, & 40 days	0, 5, 15, 25, & 40 °C	[93]	S31
Queensland, Australia	*Afourer mandarins*	1–28 days & 14–56 days	20, 10, & 5 °C	[94]	S32
Rabat, Morocco	*Moroccan mandarin*	0, 7, 14, 21, 28, 35, 42, & 49 days	3 °C	[95]	S33
Sardinia, Italy	*Minneola tangelos (hybrid)*	0–30 days	20 °C	[96]	S34
Sardinia, Italy	*Torocco, Moro,* *Sanguinello, & Doppio sanguigno oranges*	0, 16, 47, & 54 days	1, 8, & 20 °C	[97]	S35
Sargodha, Pakistan	*Kinnow mandarin*	0–84 days	4 °C	[98]	S36
Siracusa, Italy	*Torocco & Moro oranges*	0–85 days & 0–106 days	8 & 22 °C	[99]	S37
South coast, Israel	*Star ruby grapefruit*	0–35 days	10 & 20 °C	[100]	S38
South Texas, Texas	*Rio red grapefruit*	0–35 days	11 & 21 °C	[101]	S39
Spain	*Clemenules mandarin*	At harvest; 6, 9, & 12 days	1.5 °C	[102]	S40
Swat, Pakistan	*Pakistani blood red oranges*	0, 7, 14, 21, 28, 35, & 42 days	25–30 °C	[103]	S41
Taichung, Taiwan	*Murcott tangor (hybrid)*	56 days	15 °C	[104]	S42
Tanga, Tanzania	*Msasa & Jaffa oranges*	0, 4, 8, & 12 days	18 & 28 °C	[105]	S43
Texas	*Rio red grapefruit*	0 & 35 days	10 °C (28 days) & 20 °C (7 days) [106]	[107]	S44
Texas, US	*Rio red grapefruit*	0–28 days	9 & 23 °C	[106]	S45
Turkey	*Satsuma mandarin*	0–28 days	20 °C	[108]	S46
Valencia, Spain	*Navelina oranges,* *Clemenules mandarin,* *Clemenpons mandarin,* *Oronules mandarin,* *Mutant mandarins (Prenules, Basol,* *Clemenrubí, and Orogros)*	16 days	1 °C	[43]	S47
Valencia, Spain	*Valencia oranges*	28, 56, & 112 days	5 °C	[109]	S48
Valencia, Spain	*Fortune, Nova,* *Nadorcott mandarin*	0, 21, & 56 days	2 °C	[110]	S49
Valencia, Spain	*Valencia oranges*	0, 10, 15, 20, & 40 days	4 °C	[111]	S50
Xiangtan, China	*Satsuma mandarin*	0–8 days	25 °C	[112]	S51
Xiangtan, China	*Ponkan mandarin*	0, 3, & 6 days	25 °C	[113]	S52
Yilan orchard, Taiwan	*Tankan mandarin*	0 & 42 days	13 °C	[114]	S53
Zhejiang, China	*Satsuma mandarin*	60 days	10 °C	[115]	S54

### 2.3 Publication bias and quality assessments

**Fig. 2:**
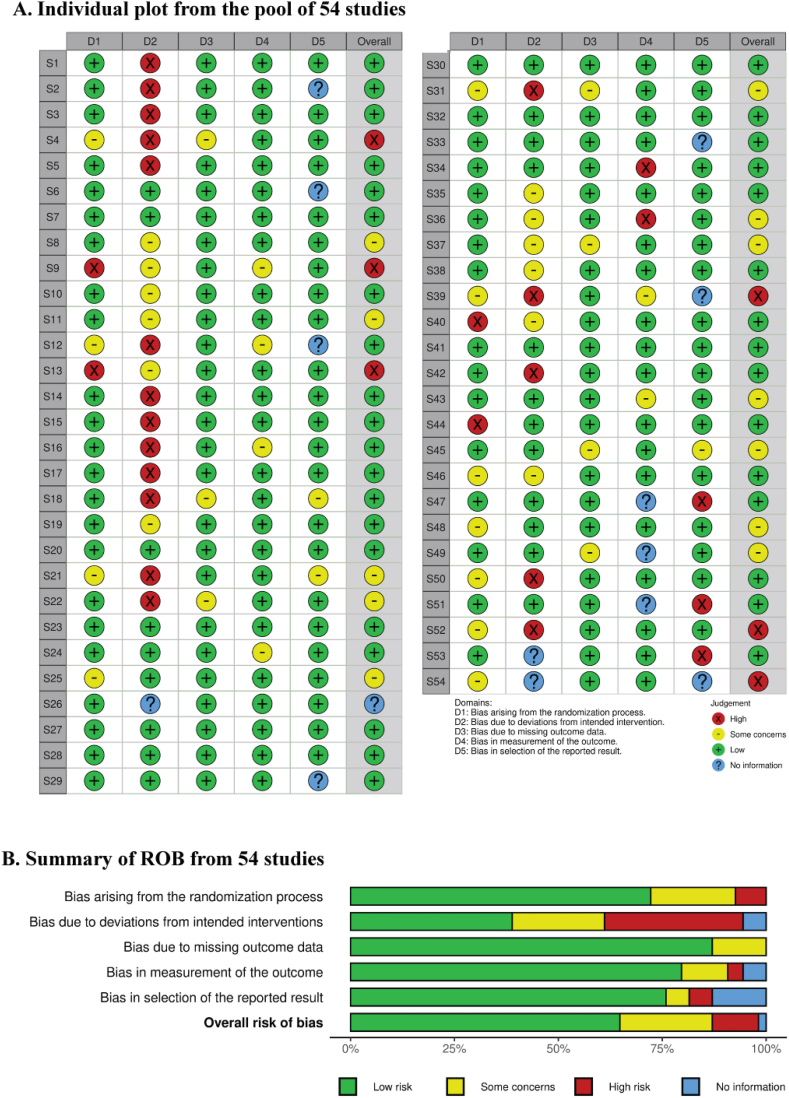
The risk of bias (ROB) analysis of 54 selected studies, which served as the data source for the meta-analysis on the influence of temperature and duration of storage on the quantity of vitamin C in citrus fruits. Individual ROB (A) and Summary of ROB Across Studies (B).(1)$Y_{ij}=\mu +s_{i}+\tau_{j}+{s\tau }_{ij}+\beta_{0}+\beta_{1}L_{ij}+b_{i}L_{ij}+\beta_{2}{L_{ij}}^{2}+{b_{i}L_{ij}}^{2}+e_{ij}$

The study limitations, also known as the risks of bias inherent to overall studies, were investigated using the Cochrane collaboration assessment method [62]. A total 54 selected used in this study asses the individual bias (Fig. 2a.). This assessment involved evaluating various criteria, such as the duration of storage and storage temperature of citrus fruits was evenly distributed (D1), identifying deviations from temperature and duration of storage interventions on the concentration of vitamin C in citrus fruits (D2), the presence of missing data on the values of vitamin C concentration outcomes (D3), how each researcher measured the validity of storage method and storage temperature against the level of vitamin C in citrus fruits (D4), and the researchers subjectivity in reporting the results of vitamin C concentration (D5). Two independent researchers conducted this evaluation. In order to meet each criterion was judged hierarchically, with a “low risk” assigned a score of 3, “some concerns” given a score of 2, and “high risk” given a score of 1. These scores were then used to calculate an overall risk of bias for each study. The individual assessments for each criterion were summarized in a table and inputted into the Robvis (Risk-of-Bias VISualization) website to generate traffic light plots and weighted bar plots [63]. The summary risk of bias is depicted in Fig. 2b.

### 2.4 Meta-analysis and its model

A meta-analysis was conducted to determine the variation of vitamin C due to temperature treatment and storage duration using the linear mixed model (LMM) method [57]. In this study, the vitamin C levels were considered as the fixed factor, while the variability in individual study results was treated as the random factor. Validation tests were performed using *P* values (from analysis of variance or ANOVA), residual mean square error (RMSE), and determination coefficient (R^2^). Additionally, Tukey test was employed to analyze categorical data. Furthermore, changes in temperature and storage duration of vitamin C were analyzed using a regression (meta-regression) as graph visualization. The following earlier studies [57,58] were employed in the meta-analysis model, with the mathematical equation in Eq. (1).

Note: $Y_{ij}$ – a dependent variable, μ- overall mean value, $s_{i}$ – random effect from the i-^th^ study, with an assumption of $∼\;N_{iid}\;\left(0,\sigma_{S}^{2}\right)$, $\tau_{j}$ – the fixed effect of the j-^th^ level of τ factor, ${s\tau }_{ij}$ – a random interaction between the i-^th^ study and the j-^th^ level of τ factor, with an assumption of $∼\;N_{iid}\;\left(0,\sigma_{S\tau }^{2}\right)$, $\beta_{0}$ is the value of the intersection of the average of all studies with the axis, $\beta_{1}$ is the regression coefficient, $L_{ij}$ and ${L_{ij}}^{2}$ are the number of predictor in linier and quadratic, as storage time (day) and storage temperature (°C), $b_{i}$ is the random effect of the difference in studies from the regression coefficient Y in the X of the i-th study, and $e_{ij}$ is the unexplained error value.

### 2.5 Software, statistical analysis, and response surface method

The software utilized for analysis is R version 4.2.0, supplemented with additional packages including lme4 for data modeling, lmerTest for categorical testing, and performance for validation testing with a 5 % error threshold using Tukey test [116]. Validation data analysis of RMSE (Eq. (2)) and R^2^ (Eq. (3)) were carried out in R [117,118].

(2) $$RMSE=\sqrt{\frac{\sum {\left(A-E\right)}^{2}}{n}}$$

(3) $$R^{2}=\frac{\left(s_{f}^{2}+\sum \left(s_{l}^{2}\right)\right)}{\left(s_{f}^{2}+\sum \left(s_{l}^{2}\right)+s_{e}^{2}+s_{d}^{2}\right)}$$

where A = real data, E = estimated data, n = number of data, $s_{f}^{2}$ is the variant of a fixed factor, $\sum \left(s_{l}^{2}\right)$ is the sum of all variants of the component, $s_{e}^{2}$ is the variant due to the predictor dispersion, and $s_{d}^{2}$ is the specific distribution of the variant. Then, to measure the significance of the model, a variance analysis test was carried out, which is significant if *P* ≤ 0.05 and tended to be significant if *P* ≤ 0.1. Then, a Tukey test is conducted to determine the influence of citrus genotype, storage time (day), and storage temperature (°C) on the vitamin C level in mg/100 mL [119,120].

In addition, a response surface methodology (RSM) is applied to measure the influence of factor A (storage time, day) and factor B (storage temperature, °C) on the vitamin C (mg/100 mL) content of citrus (the response). Optimization is also employed to find the optimal values for factors A and B [121]. The creation of the RSM and optimization is carried out using Design Expert “Version 13”.

## 3 Results and discussion

### 3.1 General outlook of database used for meta-analysis

Numerous data points were harvested from 54 single studies on citrus vitamin C in response to different origins, genotypes, storage times, and temperatures. The number of data points on vitamin C in the present meta-study was 1,402, with the mean and maximum value of vitamin C at about 32.2 and 76.2 as mg/100 mL, respectively (Table 3). Several data points in terms of storage time and storage temperature were 1412 and 1385 points, respectively. Concerning citrus species, there were variations in vitamin C data points, i.e., 669 points in mandarin, 451 points in orange, 129 points in lime, 127 points in grapefruit, 17 points in hybrid citrus, 10 points in tangerine and 9 points in lemon. Descriptively, the highest vitamin C was found in the maximum value of lemon species, for about 76.2 mg/100 mL (Table 3). The discrepancy among numerous single studies commonly occurred during the initial step of database construction for meta-analysis study. Those discrepancies need to be studied further using meta-analysis, because meta-analysis could produce stronger statistical power, combine more single studies to form a bigger sample size, and obtain a comprehensive summary in a time-efficient way. Several published studies have reported the use of meta-analysis on citrus, such as to summarize the relationship between fruit intake and cancer risk [[122], [123], [124]]. Huanglongbing resistance gene identification [125], varietal selection [126], and the water and nitrogen use efficiencies [127].

**Table 3:** Descriptive statistics of the meta-analysis data of vitamin C content in citrus fruits.

Variable	NDP	Mean	SD	Max	Min	Q25	Q50	Q75
Whole data								
Storage Time (days)	1412	29.1	3.46	330	0	7	18	40
Storage Temperature (°C)	1385	13.6	9.89	40	0	4	10	25
Vitamin C (mg/100 mL)	1402	32.2	16.2	76.2	0	20	32.2	43.1
Citrus genotype								
Grapefruit								
Storage Time (days)	127	16.6	2.32	112	0	1	5	28
Storage Temperature (°C)	127	12.4	6.61	23	4	9	11	20
Vitamin C (mg/100 mL)	127	33.3	9.88	64.8	11.7	26.3	34.6	39.5
Hybrid								
Storage Time (days)	17	35.8	21.4	56	0	10	30	56
Storage Temperature (°C)	17	17.7	2.57	20	15	15	20	20
Vitamin C (mg/100 mL)	17	48.3	18.7	75.5	29.6	33	34.5	64.1
Lemon								
Storage Time (days)	9	60	26	90	30	30	60	90
Storage Temperature (°C)	9	10	–	10	10	10	10	10
Vitamin C (mg/100 mL)	9	65.5	5.38	76.2	59.3	61	64.2	68
Lime								
Storage Time (days)	129	22.2	20.4	90	0	12	18	24
Storage Temperature (°C)	129	22.2	8.49	27.5	8	10	27.5	27.5
Vitamin C (mg/100 mL)	129	31.6	6.13	49.9	16	29	30.9	34.7
Mandarin								
Storage Time (days)	669	26	24.4	120	0	8	18	42
Storage Temperature (°C)	669	12.4	9.56	25	1	4	6.5	25
Vitamin C (mg/100 mL)	659	25.7	15.8	67.7	1.7	12.8	21.2	38.6
Orange								
Storage Time (days)	451	38.9	48.7	330	0	8	20	60
Storage Temperature (°C)	424	13	10.6	40	0	5	6.5	24
Vitamin C (mg/100 mL)	451	40.5	15.4	73.5	0	32.3	41.7	50.9
Tangerine								
Storage Time (days)	10	7	–	7	7	7	7	7
Storage Temperature (°C)	10	20	–	20	20	20	20	20
Vitamin C (mg/100 mL)	10	16.9	2.34	20.9	13.2	15.9	16.5	17.8

Quantile statistics are expressed as follows: Q25 – 25 %, Q50 – 50 %, and Q75 – 75 %; Max – maximum the feature data value; Min – Minimum the feature data value; NDP – Number of data points; SD – Standard deviation.

### 3.2 Meta-regression and optimum condition of vitamin C in different citrus species and storage treatment

**Fig. 3:**
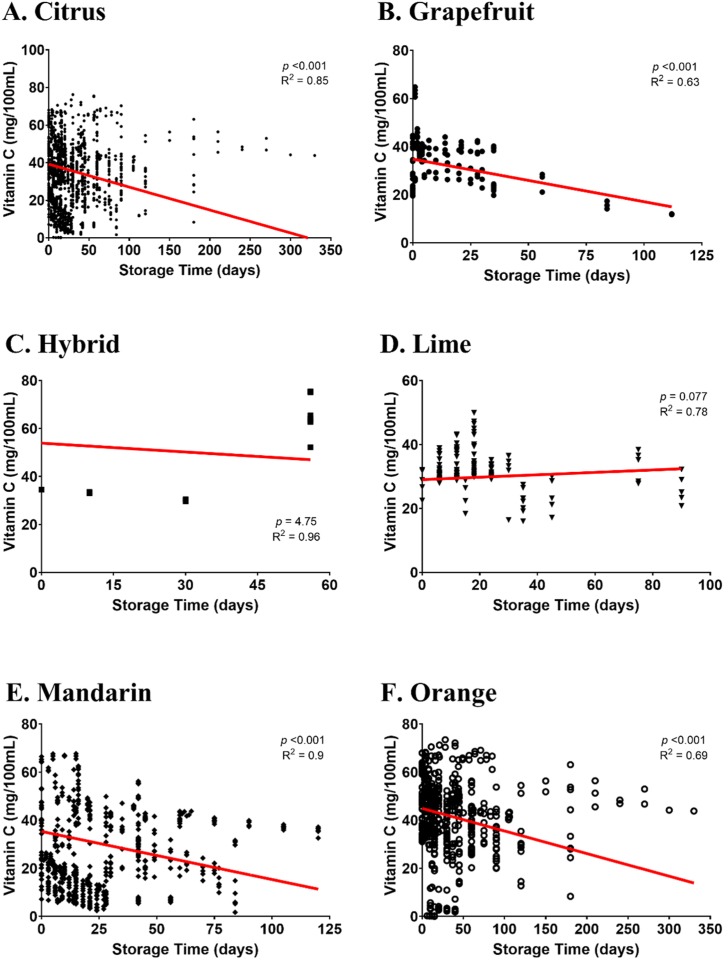
Regression of citrus genotype due to storage time (days) across six varieties of citrus: citrus (A), grapefruit (B), hybrid (C), lime (D), mandarin (E), and orange (F). The regression values (indicating a strong correlation if R^2^ > 0.7 [128]) and significance (considered significant if *P* ≤ 0.05) of vitamin C across various types of citrus fruits in relation to storage duration (days) are assessed. (For interpretation of the references to colour in this figure legend, the reader is referred to the Web version of this article.)

**Fig. 4:**
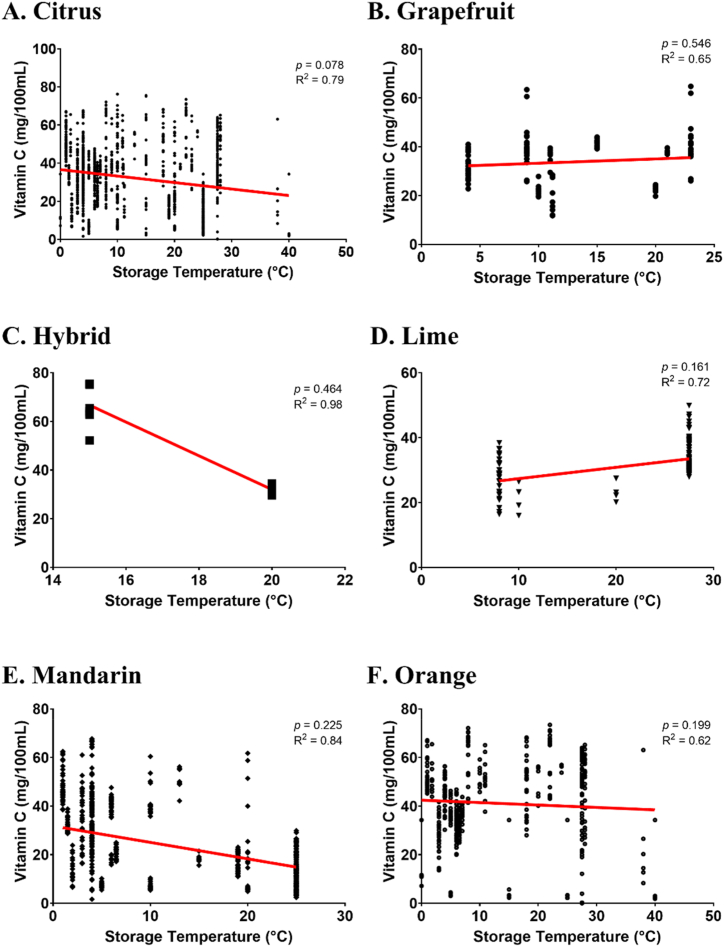
Regression analysis of citrus genotype influenced by storage temperature (°C), i.e., citrus (A), grapefruit (B), hybrid (C), lime (D), mandarin (E), and orange (F). The study evaluates the correlation strength (considered strong if R^2^ > 0.7 [128]) and significance (deemed significant if *P* ≤ 0.05) of vitamin C in different citrus fruits concerning storage temperature (°C). (For interpretation of the references to colour in this figure legend, the reader is referred to the Web version of this article.)

The present study revealed meta-regression result of vitamin C in different citrus species and storage time (Table 3) and storage temperature (Table 4). Quantitative meta-regression on 1402 data points revealed that there was significant effect of storage time on citrus vitamin C and showed a negative trend on both linear and quadratic models (Table 4). Concerning grapefruit, mandarin, and orange, there was a significant effect of storage time on their vitamin C level. The forming regression was a negative and linear pattern. In contrast, there was no significant meta-regression found in terms of vitamin C level in hybrid citrus and lime as the impact of storage time. In Fig. 3, all citrus genotypes exhibit a confirmed decrease in vitamin C levels, except for limes, which show an increase in quantity. An analysis using quantitative meta-regression with 1375 data points indicated that storage temperatures did not have a significant impact on the vitamin C content of citrus fruits in most cases. However, hybrid citrus fruits did show a significant decrease (*P* < 0.001) in the quadratic model (see Table 5). It is worth noting that the overall data trended downwards, although this trend was not statistically significant (*P* = 0.078). As seen in Fig. 4, the decline in vitamin C is attributed to the rise in storage temperature, but this is not the case for grapefruit and lime varieties.

**Table 4:** Meta-regression of vitamin C levels on several Citrus species in response to different storage times.

Variable	M	NDP	Intercept		Slope		Validation		
			Value	SE	Value	SE	*P* value	RMSE	R^2^
Whole Data	L	1402	39.4	2.27	−0.122	0.009	<0.001	6.99	0.85
	Q		40.3	2.26	−0.194	0.014	<0.001	6.89	0.85
					0.00049	0.00008	<0.001		
Citrus genotype									
Grapefruit	L	127	34.8	3.1	−0.114	0.031	<0.001	5.86	0.63
	Q		33.6	3.13	0.029	0.065	0.653	5.72	0.64
					−0.00168	0.00067	0.014		
Hybrid	L	17	53.9	20.9	−0.123	0.168	0.475	5.26	0.96
	Q		44	14.2	−0.83	0.583	0.542	5.39	0.92
					0.02	0.0146	0.675		
Lime	L	129	29	3.78	0.038	0.021	0.077	3.86	0.78
	Q		27.5	4.08	0.161	0.063	0.012	3.79	0.81
					−0.00144	0.0007	0.041		
Mandarin	L	659	35.3	3.52	−0.2	0.012	<0.001	5.52	0.9
	Q		37.1	3.56	−0.344	0.03	<0.001	5.4	0.91
					0.00161	0.0003	<0.001		
Orange	L	451	44.9	3.07	−0.094	0.016	<0.001	9.02	0.69
	Q		46.6	3.05	−0.19	0.028	<0.001		
					0.00048	0.00012	<0.001		

L – Linear; Q – Quadratic; M − Model; NDP – Number of data point; R^2^ – r determinant value; RMSE – Residual mean square error; SE – Standard error.

**Table 5:** Meta-regression of vitamin C levels on several Citrus species in response to different storage temperatures.

Variable	M	NDP	Intercept		Slope		Validation		
			Value	SE	Value	SE	*P* value	RMSE	R^2^
Whole Data	L	1375	36.7	2.21	−0.088	0.05	0.078	7.5	0.79
	Q		36	2.36	0.043	0.164	0.794	7.49	0.79
					−0.00398	0.00476	0.403		
Citrus genotype									
Grapefruit	L	127	31.5	3.83	0.072	0.119	0.546	6.13	0.65
	Q		31	15.8	0.152	2.351	0.95	6.13	0.68
					−0.00257	0.0746	0.974		
Hybrid	L	17	171	163	−6.94	9.24	0.464	5.4	0.98
	Q		171	9.24	−6.94	15	<0.001	5.4	0.98
					163	15	<0.001		
Lime	L	129	24	5.14	0.321	0.217	0.161	3.91	0.72
	Q		36.8	11	−2	1.76	0.299	3.9	0.74
					0.071	0.0528	0.246		
Mandarin	L	659	31.8	3.44	−0.12	0.099	0.225	6.61	0.84
	Q		32.2	3.98	−0.218	0.565	0.701	6.61	0.84
					0.00367	0.021	0.861		
Orange	L	424	42.5	3.14	−0.099	0.077	0.199	9.58	0.62
	Q		41.5	3.3	0.1	0.245	0.683	9.58	0.61
					−0.00568	0.00663	0.392		

L – linear; Q – quadratic; M − model; NDP – number of data point; R^2^ – r determinant value; RMSE – residual mean square error; SE – standard error.

The deterioration of horticultural products due to their shelf life is indeed a particular concern. Active ingredients such as vitamins and volatile compounds are often subject to denaturation and reduction in quantity [74,77,78,100,101,106,108]. Consistent with the findings from this meta-regression, it is observed that citrus fruits will experience a decrease in the quantity of ascorbic acid if stored for an extended period. The gradual degradation of vitamin C amounts to 0.122 mg/100 mL per day, and it is estimated that the complete deterioration of ascorbic acid will occur on the 323^−rd^ day after postharvest [93]. Therefore, some researchers are actively engaged in environmental engineering efforts (such as the use of active packaging) or the application of advanced technologies (such as long-wave irradiation and ionization) to mitigate the degradation of ascorbic acid due to the latent heat properties of the material [129].

The general analogy concerning material damage due to temperature increase is inevitably bound to occur. Similarly, the vitamin C content in citrus fruits will experience a decrease in concentration when exposed to high temperatures. All types of citrus fruits undergo ascorbic acid damage at a rate of less than 0.321 mg/100 mL per day, except for the hybrid varieties at 6.94 mg/100 mL each day. Hybrid varieties such as Murcott Tangor and Minneola Tangelos excel in terms of quality but tend to have lower resistance to damage and decay [96,104]. On the other hand, grapefruits and limes are resistant to high storage temperatures of up to 23 and 27.5 °C [84,107].

**Fig. 5:**
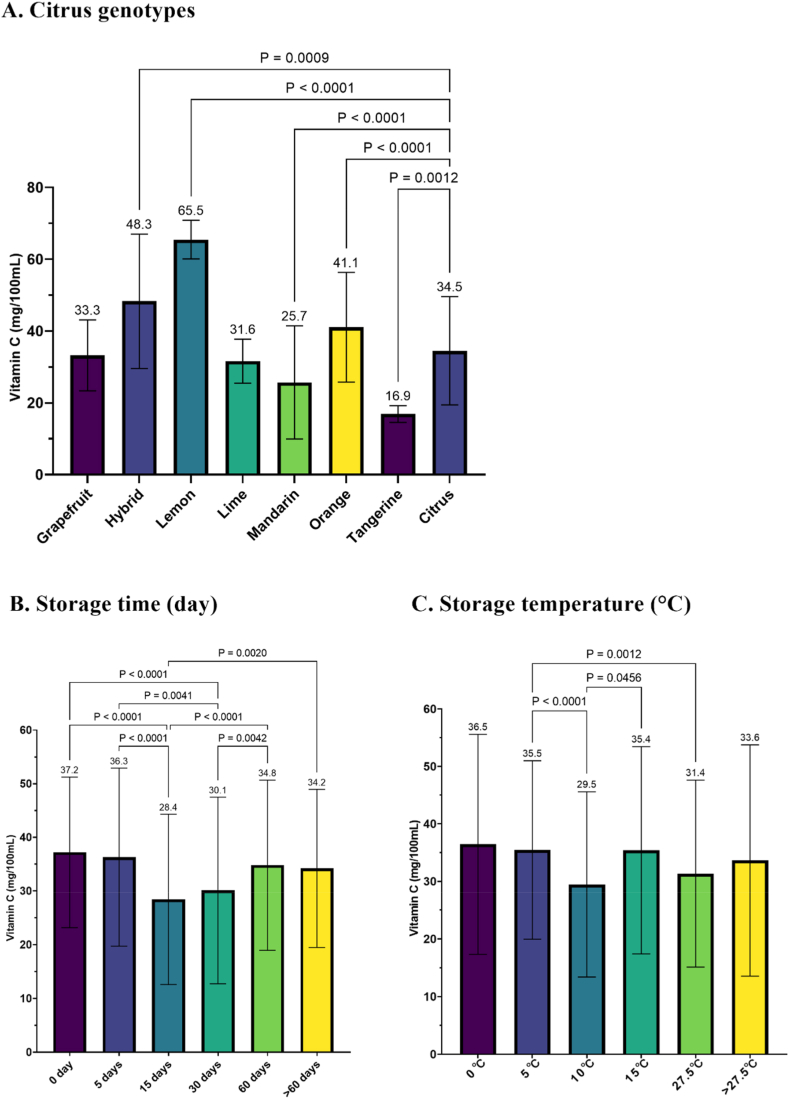
Differences in vitamin C levels (mg/100 mL) among various citrus genotypes (A), the impact of storage duration (B), and storage temperature (C). There is a 5 % variation in the Tukey test.

The vitamin C content of citrus fruits is directly affected by both storage temperature and storage duration. Nevertheless, some citrus varieties exhibit resistance to changes in storage temperature and storage duration. For example, oranges appear to be less susceptible to alterations in storage temperature with regard to their vitamin C content [72,93]. A meta-regression analysis suggests that this resistance to storage duration applies to “Kagzi Acid Lime” as well [84]. The recorded changes from the meta-regression model indicate a gradual increase in vitamin C of 0.038 mg/mL per day (*P* = 0.77) over a period of 90 days, resulting in a residual vitamin C content of 49.9 mg/100 mL. Various citrus genotypes exhibit differences in their vitamin C content due to variations in storage times and temperatures (Fig. 5a). Tangerine had significantly lower vitamin C content than other citrus genotypes (16.9 mg/100 mL; *P* < 0.001). The two highest vitamin C content were found in lemon and hybrid citrus (*P* < 0.001), around 65.6 and 48.3 mg/100 mL, respectively.

The variation in storage temperature did not yield a noteworthy impact on the vitamin C levels in citrus when compared to the control temperature of 0 °C (Fig. 5c). Thus, high storage temperatures accelerated the decomposition of vitamin C in citrus fruit, as reported in previous studies [53,54]. This is evident from the significant decrease (*P* ≤ 0.05) in vitamin C levels at temperatures of 10, 15, and 27.5 °C, which occurred at 29.5, 35.4, and 31.4 mg/100 mL, respectively. In the study, the time of storage had a notable impact on the vitamin C content in citrus (see Fig. 5b). As the storage time increased, the oxidation of ascorbic acid became more pronounced [104,106,109,114]. In general, the vitamin C content significantly decreased to 30.1 mg/100 mL after storage for more than 30 days (*P* < 0.001). However, storage for more than 60 days (34.2 mg/100 mL) showed no statistically significant change compared to the control (*P* > 0.05). Further confirmation is necessary, but it is suspected that citrus genotype and room storage temperature may have an influence (as seen in limes, which tend to experience an increase in vitamin C content during storage). Additionally, further confirmation through RSM is needed to understand the interaction conditions and optimization between temperature and storage duration.

**Fig. 6:**
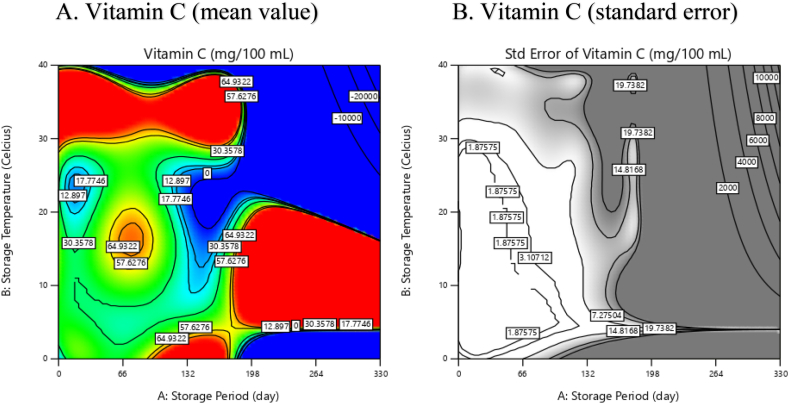
The response surface of the relationship between storage temperature and duration concerning the vitamin C content of citrus fruits, in mean value (A) and standard error (B).

**Fig. 7:**
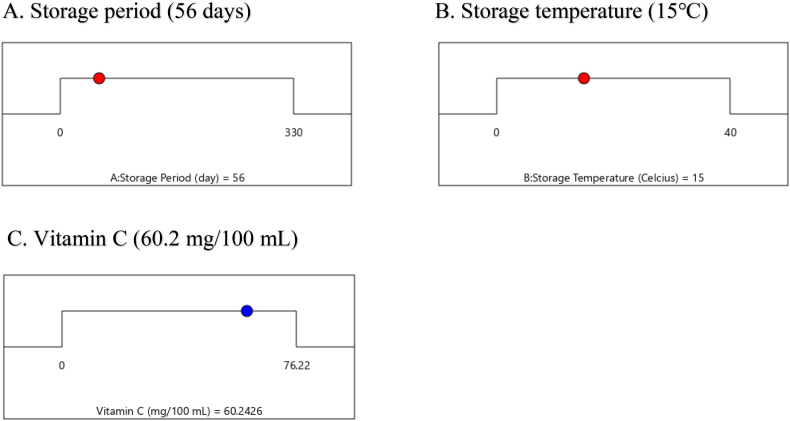
The effect of Factor A (storage time, A) and Factor B (storage temperature, B) to optimize vitamin C content (C) in citrus during storage.

In general, lower temperatures are more effective in preserving vitamin C, while vitamin C tends to degrade more rapidly at ambient (higher) temperatures [70,75,85,93,94,110]. There exists an optimal temperature range for storing citrus fruits to minimize vitamin C loss, and excessively low temperatures can also lead to vitamin C deterioration. For instance, “Blood Red” sweet oranges stored at 10 °C retained a higher amount of vitamin C compared to those stored at 5 °C or 20 °C [103]. The results from RSM and meta-analysis confirm that the ideal temperature range for citrus storage to minimize vitamin C loss is between 10 and 20 °C (Fig. 6). However, temperature control alone is not sufficient; humidity regulation becomes a crucial factor. Maintaining a high relative humidity of 90–95 % during storage is essential to preserve the quality of fresh produce because lower RH levels can lead to increased transpiration rates and a faster deterioration in external quality [21]. Optimization results from the response surface methodology indicate that storing citrus at 15 °C for 56 days can maintain citrus vitamin C content at 60.2 mg/100 mL (Fig. 7).

## 4 Conclusion

In conclusion, storage duration and temperature are indeed the primary factors contributing to a decline in the chemical quality of citrus, especially the level of vitamin C. To address this, keeping the temperature at 15 °C and limiting the storage time to a maximum of 56 days can maintain the concentration of vitamin C at a stable level of 60.2 mg/100 mL. In detail, the genotype factor of citrus fruits results in lemons and hybrids having the highest vitamin C content, while tangerines have the lowest.

## Declaration of the competing interest

The authors assert the absence of any disclosed financial conflicts of interest or personal relationships that might have been perceived to influence the research reported in this paper. This research was funded by 10.13039/501100015690Universitas Padjadjaran, Indonesia through the scheme of library and online data research grant, number 2203/UN6.3.1/PT.00/2022. The APC was also provided by Universitas Padjadjaran, Indonesia.

## Data availability statement

The authors confirm that the data supporting the findings of this study are available within the article upon reasonable request.

## CRediT authorship contribution statement

**Rahmat Budiarto:** Writing – review & editing, Writing – original draft, Supervision, Project administration, Conceptualization. **Syariful Mubarok:** Resources, Investigation, Data curation. **Mohammad Miftakhus Sholikin:** Writing – review & editing, Writing – original draft, Visualization, Software, Formal analysis. **Dwi Novanda Sari:** Resources, Investigation, Data curation. **Ana Khalisha:** Resources, Investigation, Data curation. **Stefina Liana Sari:** Resources, Investigation, Data curation. **Bayu Pradana Nur Rahmat:** Resources, Investigation, Data curation. **Tri Ujilestari:** Writing – review & editing, Validation, Methodology, Investigation, Formal analysis. **Danung Nur Adli:** Writing – review & editing, Methodology.
